# Evaluation of diagnostic concordance between algorithms for Parkinson’s disease dementia

**DOI:** 10.1177/1877718X261450361

**Published:** 2026-06-11

**Authors:** Martina Mana, Josef Mana, Petra Stofanikova, Tereza Uhrova, Robert Jech, Ondrej Bezdicek

**Affiliations:** 1 Department of Neurology and Centre of Clinical Neuroscience, First Faculty of Medicine and General University Hospital in Prague, Charles University, Czech Republic

**Keywords:** Parkinson’s disease, dementia, diagnostic criteria, functional assessment, cognitive assessment

## Abstract

**Background:** Significant number of patients with Parkinson’s disease (PD) gradually progress to Parkinson’s disease dementia (PDD). Recent proposal for updating current diagnostic criteria for PDD recommends alternative screening tests and broader functional assessments. **Objective:** To evaluate the diagnostic concordance among algorithms for PDD based on Level I (i.e., screening) criteria and to assess their predictive validity for Level II (i.e., neuropsychological battery) diagnosis. **Methods:** A cross-sectional retrospective analysis of 190 patients with PD who underwent a comprehensive neuropsychological assessment. A total of 68 diagnostic algorithms were operationalized using combinations of scores derived from the Mini-Mental State Examination (MMSE) and the Montreal Cognitive Assessment (MoCA). Functional impairment was based either on the Functional Assessment Questionnaire (FAQ) item 9 or the FAQ total score. Diagnostic concordance was evaluated using Cohen’s κ. Predictive validity for Level II classification was assessed using projection predictive variable selection. **Results:** Estimated PDD rates ranged from 2.1% up to 16.8%. Concordance was moderate to high among algorithms using the same functional impairment definition (κ_FAQ total_ = 0.75, κ_FAQ 9_ = 0.86) but substantially lower when functional impairment definitions differed (κ = 0.43). A parsimonious screening model combining MoCA Five Words and MMSE Sevens adequately approximated Level II classification and yielded a prevalence-adjustable heuristic decision rule. **Conclusions:** Diagnostic outcomes for PDD are sensitive to the choice of cognitive and functional instruments suggesting that different algorithms may capture partially distinct constructs. Minimal screening using selected items can approximate Level II PDD diagnosis, but this simplification entails a trade-off between sensitivity and specificity.

## Introduction

Parkinson’s disease (PD) is a neurodegenerative disorder typically characterized by a progressive onset of motor symptoms, including rigidity, bradykinesia, postural instability and resting tremor. Moreover, patients suffer from a range of non-motor impairments,^1^ particularly cognitive decline. This factor might result in Parkinson’s disease dementia (PDD) in a subset of patients.^2^

According to a recent meta-analysis, approximately one-quarter of PD patients are likely to be diagnosed with PDD.^3^ However, reported PDD rate estimates vary widely, ranging from 14% up to 55%, depending on the methodological criteria employed. Moreover, factors such as patients’ sex,^4^ age and disease duration appear to modulate the risk of cognitive decline and PDD.^5,6^

Despite the clinical relevance of PDD, its diagnosis remains complex. A milestone in research of PDD was the publication of diagnostic criteria established in 2007 by the International Parkinson and Movement Disorder Society (MDS).^7^ In these criteria, the MDS introduced a two-levelled system for PDD detection. Level I consists of brief cognitive assessments, while Level II involves comprehensive neuropsychological testing across cognitive domains.^8^

The original Level I algorithm included eight conditions that had to be satisfied simultaneously in order to diagnose probable PDD. These included: 1) diagnosis of PD proposed by the Queen Square Brain Bank; 2) PD onset prior to the PDD emergence; 3) evidence of global cognitive impairment (MMSE score < 26 points); 4) cognitive deficit interference with the IADL (assessed by the pill questionnaire or caregiver interview); 5) impairment in at least two cognitive domains, namely memory, attention, visuo-constructive abilities and executive function; 6) there was absence of Major Depressive Disorder; 7) absence of delirium; and 8) exclusion of other abnormalities and potential causes of dementia.^7^

Currently, efforts are focused on refining this PDD diagnostic framework. A recent call for a change pinpoints limitations regarding the original criteria and suggests various updates to enhance their utility.^9^ Proposed suggestions include replacement of the Mini-Mental State Examination (MMSE) by the Montreal Cognitive Assessment (MoCA), which is more sensitive to PD-specific cognitive impairment; expansion of instrumental activities of daily living (IADL) evaluation; inclusion of language assessment; recognition of anxiety as one of the neuropsychiatric symptoms relevant in PDD; and integration of biomarkers.

In light of these proposals, the current study aims to evaluate the diagnostic concordance between the original MDS Level I PDD criteria^7,8^ and a modified framework based on the recent call for change.^9^ Furthermore, both Level I diagnostic approaches are compared to PDD diagnosed on Level II. The study aims to address the following research objectives (RO): (RO1) To estimate the PDD rate and evaluate the diagnostic variability and concordance across different PDD criteria. (RO2) To identify components of the diagnostic criteria contributing to PDD classification variability across the applied criteria. (RO3) To explore the accuracy of Level I screening criteria in predicting Level II PDD classification.

## Methods

### Participants

This study retrospectively analyzed clinical data from a cohort of patients with PD at the General University Hospital in Prague. All patients were diagnosed with idiopathic PD by a movement disorder specialist according to the MDS Clinical Diagnostic Criteria for PD.^1^ Clinical records spanning January 2015 to February 2025 were examined. All participants were candidates for Deep Brain Stimulation (DBS) treatment and underwent neuropsychological evaluation conducted by a trained clinical psychologist (OB) as part of standard preoperative assessments for DBS eligibility at the General University Hospital in Prague.

Consequently, patients with neurological or psychiatric disorders other than PD, including atypical Parkinsonism, the use of anticholinergic medication, neurological conditions potentially resulting in cognitive impairment, e.g., stroke, psychoactive substance abuse, epileptic seizures, or gait disorders unresponsive to optimal dopaminergic treatment, were excluded from the study. Patients with tremor-dominant PD, rather than predominance of axial symptoms, and better response to dopaminergic treatment were considered preferentially. Nonetheless, axial symptoms such as hypokinetic dysarthria, postural instability and gait freezing/hesitations were not used as hard exclusion criteria.

### Neuropsychological assessment

Cognitive performance was evaluated at both Level I and Level II according to the standard MDS battery for Parkinson’s Disease Mild Cognitive Impairment (PD-MCI).^10,11^ All neuropsychological assessments were conducted while patients were in the ON state of medication (usual medication dose). Cognitive performance at Level I was assessed by the MMSE^12,13^ and the MoCA.^14,15^ The comprehensive neuropsychological assessment at Level II evaluated five cognitive domains through specific tests: attention and working memory assessed by Trail Making Test Part A (TMT-A),^16,17^ and WAIS Digit Span Backward (WAIS DSB),^18^ executive function by Categorical Verbal Fluency - Animals (CF-A),^19^ and subtest from the Prague Stroop Test – Colors (PST-C),^20^ language by the WAIS Similarities subtest,^18^ and the Boston Naming Test (BNT-60),^21,22^ memory by the Rey Auditory Verbal Learning Test (RAVLT-DR)^23–25^ delayed recall, and the Brief Visuospatial Memory Test–Revised (BVMTR-DR)^26,27^ delayed recall, or WAIS Family Pictures subtest^18^ delayed recall, visuospatial function assessed by the Judgment of Line Orientation Test (JoL),^28^ and Clock Drawing Test (CLOX-I).^29^

The Functional Activities Questionnaire (FAQ)^30,31^ was administered to assess functional impairment. The Beck Depression Inventory-II (BDI-II)^32,33^ and State-Trait Anxiety Inventory (STAI)^34,35^ were used to assess neuropsychiatric status.

### Diagnostic algorithms for probable parkinson’s disease dementia

In this study, we applied three distinct sets of diagnostic algorithms for probable PDD at Level I. The first set was based on the original framework,^7^ which utilized the MMSE as a global cognitive screening tool, supplemented by assessments of attention, executive function, visuospatial abilities, and memory. The second set of algorithms was based on the recent call for change of dementia diagnostic guidelines,^9^ which advocates for more sensitive cognitive domain assessments in the context of PD. This updated approach incorporated specific items from the MoCA. The third approach applied the Czech version of the shortened Montreal Cognitive Assessment (sMoCA),^36^ a time-efficient modification designed to measure global cognitive performance using a reduced testing protocol that omits items providing redundant information. The sMoCA has been validated in the Czech PD cohort^36^ and shown to be sensitive to cognitive deficits while lowering patient burden.^37^ We included the sMoCA in our study for its clinical utility in pre-surgical settings, where time restrictions and patients’ fatigue often limit the feasibility of longer assessments. Moreover, the Czech validation study reported comparable diagnostic accuracy between MoCA (AUC = 0.815) and sMoCA (AUC = 0.796) for distinguishing PD-MCI from PD-NC, supporting the sMoCA as a suitable and efficient alternative.

Lastly, the fourth approach followed the Level II protocol for diagnosis of PDD and PD-MCI.^7,10^ The Level II methodology, including the use of a regression-based normative scoring approach, has been detailed in a prior study.^11^ In this study, the thresholds for cognitive impairment at Level II were set at *z* ≤ −1.5. All non-cognitive criteria of probable PDD (i.e., diagnosis of PD that developed before dementia and absence of Major Depression, delirium or other abnormalities that obscure diagnosis) held true for all patients in the sample according to the psychiatric and neurological examinations.

**Figure 1.**
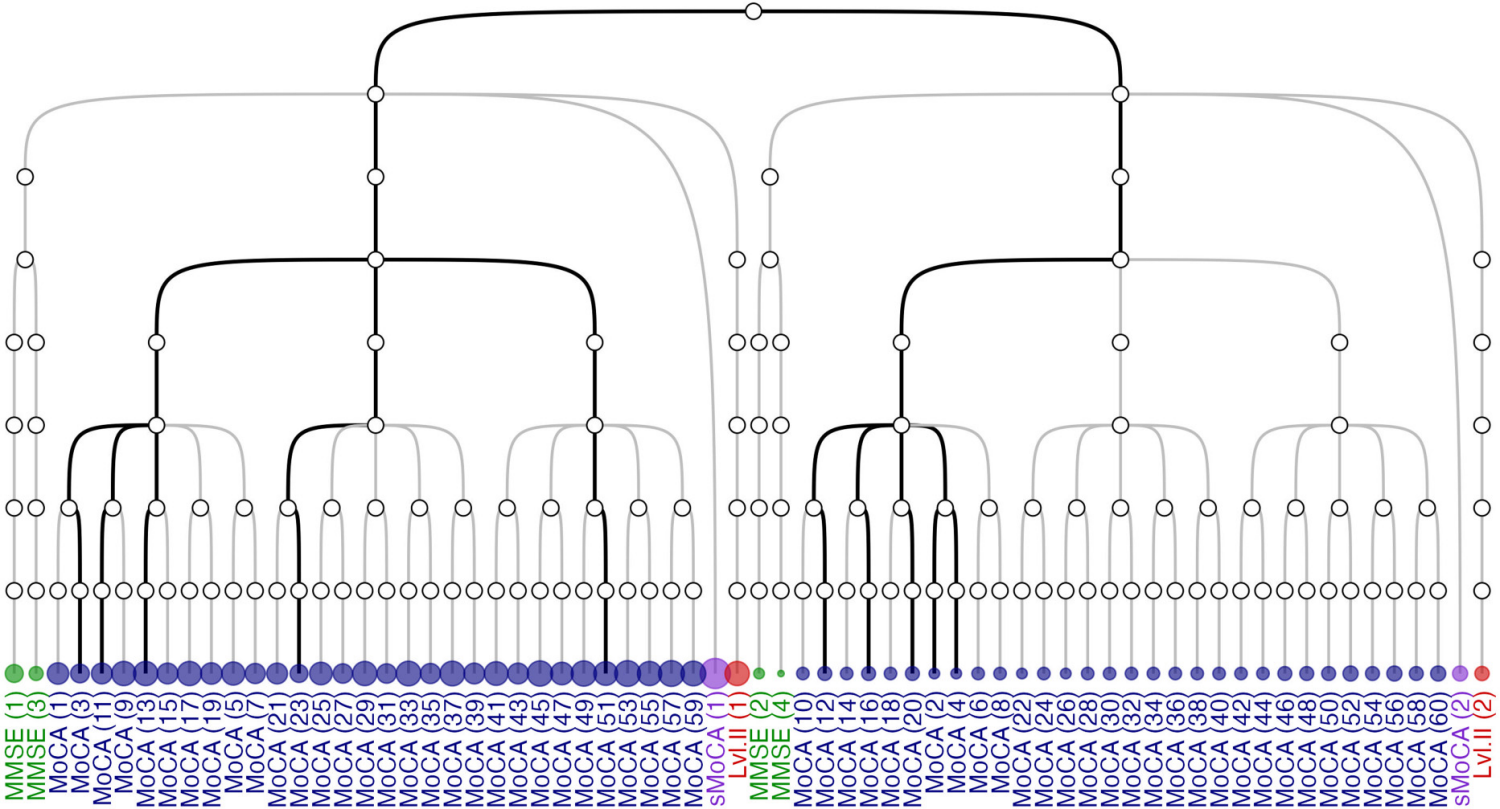
A dendrogram representing algorithms for probable Parkinson’s Disease Dementia (PDD) construction process. The dendrogram illustrates the decision process used to construct algorithms for probable Parkinson’s Disease Dementia (PDD). The second level depicts the definition of instrumental activities of daily living (IADL) deficit (FAQ total > 7 on the left, FAQ item 9 > 1 on the right). The third level indicates the selection of the screening instrument (MMSE, MoCA, sMoCA, or none in the case of Level II). Lower branches represent the selection of neuropsychological tests used to define cognitive impairment in executive function, attention, memory, and language, ordered from top to bottom as depicted in the dendrogram. Algorithms based on the MMSE are shown in green, those based on the MoCA in blue, on the sMoCA in purple, and Level II algorithms in red. The top five screening algorithms according to raw accuracy for predicting diagnosis at Level II (see Table A4) are marked by bold edges. Accompanying dots indicate the estimated PDD rate for each algorithm. The individual test items comprising each algorithm are listed in Table A1.

For each of these diagnostic approaches, we applied two operationalizations of deficits in IADL. First, we utilized FAQ item 9, which approximates the pill questionnaire from the original criteria^7^ employing a cut-off score of 2 points or higher. Second, we applied the entire Functional Activities Questionnaire (FAQ) as suggested in the call for change,^9^ employing a cut-off score of 7 points based on Czech normative data.^38^ These methodologies resulted in a total of 68 algorithms, which were distributed across different diagnostic criteria: 4 MMSE-based, 60 MoCA-based, 2 sMoCA-based, and 2 based on the Level II battery (see Figure 1, Table 1 and Appendix Table A1 for the exact specification of each algorithm).

**Table 1.** Summary of probable PDD operationalizations compared in the study.

		Impaired Cognition				
Type	Global functioning	Attention	Executive Function	Construction	Memory	Language
MMSE-based	MMSE < 26	Sevens backwards < 4	Clock drawing < 2 OR Verbal fluency (S) < 10	Pentagons < 1	3-word recall < 3	-
MoCA-based	MoCA < 27	Sevens backwards < 3	Clock drawing < {2, 3} OR Verbal fluency (K) < 11	Cube drawing < 1	5-word recall < {1, 2, 3, 4, 5}	Abstraction < 2 OR Animal naming < 3
sMoca-based	sMoCA < 13	-	-	-	-	-
Level II	-	TMT A & WAIS DSB	CF A & PST C	JoLO & CLOXI	RAVLT DR & (BVMTR DR OR WMS-III Family Pictures DR)*^a^*	WAIS Similarities & BNT 60

*^a^* The visual memory was evaluated based on WMS-III Family Pictures or BVMTR depending on which test was used in the assessment. This lead to no missing values because each patient underwent assessment via one of these tests.

Note. MMSE: Mini-Mental State Examination; MoCA: Montreal Cognitive Assessment; sMoCA: short version of the MoCA; TMT-A: Trail Making Test, Part A; WAIS DSB: Wechsler Adult Intelligence Scale Digit Span, Backwards; CF-A: Categorical Verbal Fluency, Animals; PST-C: Prague Stroop Test, Colours; WAIS Similarities: Wechsler Adult Intelligence Scale, Similarities; BNT-60: Boston Naming Test; RAVLT-DR: Rey Auditory Verbal Learning Test, Delayed Recall; BVMTR-DR: Brief Visuospatial Memory Test, Delayed Recall; WMS-III Family Pictures: Wechsler Memory Scale Family Pictures; JoL: Boston Judgement of Line Orientation; CLOX-I: Clock Drawing Test. The OR operator implies that exactly one of the criteria listed is utilized within a single operationalization; the & operator implies that both criteria are used at the same time within a single operationalization; each threshold value within the set brackets {} was used to define probable PDD once in combination with all the other criteria on the same row.

Finally, all patients were systematically evaluated for the presence of neuropsychiatric symptoms, including depression, apathy, anxiety, psychosis, and delirium, by a trained neuropsychiatrist (TU) experienced in the assessment of patients with movement disorders. Because severe psychiatric symptoms form exclusion criteria for the diagnosis of probable PDD (conditions 6–8; p. 1), all patients classified as PDD were double-checked in hospital records to confirm the absence of such confounding symptoms.

### Theoretical and empirical estimands

Following the framework proposed by Lundberg et al.,^39^ in this study we explicitly connect our research objectives and their corresponding theoretical (i.e., targets of inference) and empirical (i.e., data-driven) estimands to statistical estimates. The theoretical estimand refers to a unit-specific quantity defined over a target population and represents the ideal quantity that would address the research question under optimal conditions, such as access to complete population data or perfect experimental control. In contrast, the empirical estimand corresponds to the quantity that is actually computable using the available dataset, given real-world constraints. Throughout the study, these estimands guide both the statistical analyses performed and the interpretation of the resulting estimates.

A full description of the study’s estimands and their relation to our research objectives is presented in the Appendix (see Table A2). Briefly, our first objective concerns the rate of probable PDD and its variability introduced by alternative diagnostic algorithms in the population of patients with PD undergoing evaluation for DBS. We therefore estimate the distribution of PDD rates produced by all admissible diagnostic algorithms and evaluate concordance between their classifications. Our second objective is to examine which diagnostic components drive this variability by assessing how difference in operational definitions systematically influence PDD classification.

Importantly, although the DBS candidate cohort differs from the broader PD population in several aspects, e.g., age distribution or disease subtypes, certain conclusions derived from these analyses extend beyond our specific sample. In particular, if substantial discrepancies in classification are observed between diagnostic algorithms when applied to the same patients, the results indicate that the algorithms are not measuring the same underlying construct. Such a finding would imply either poor construct validity at large or, if circumscribed only to DBS candidates, measurement invariance. In contrast, strong concordance observed within a restricted sample would count as evidence of good construct variability for the sample but would not generalize to the broader population.

Finally, our third objective evaluates the predictive validity of Level I screening for probable PDD relative to comprehensive Level II assessment. Specifically, we rank Level I algorithms according to their accuracy in predicting Level II PDD classification and develop an exploratory screening model based on a reduced subset of cognitive screening indexes. Because this predictive model depends directly on the empirical distribution of PDD classification and test scores observed in our cohort, the resulting estimates are primarily applicable to populations of DBS candidate patients rather than the broader PD population.

### Statistical analyses

#### Variability in dementia rate estimates

To address the first study objective, we started by repeatedly assigning each patient the diagnosis of probable PDD based on each PDD algorithm listed in Table 1 (see also Table A1) resulting in a 190 (patients) × 68 (algorithms) matrix where each cell indicates whether a patient (row) meets criteria for probable PDD according to an algorithm (column). PDD rate estimates were computed as $\frac{N_{PDD}}{N_{total}}$ separately for each algorithm. The predictive value of age and sex was then evaluated by fitting a set of logistic regressions, one for each algorithm for probable PDD, whereby the probable PDD was predicted by age, sex and their interaction.

#### Concordance between diagnostic algorithms

To evaluate the second study objective, a set of two class cross-tabulations with associated statistics was computed for each pair of algorithms via the **confusionMatrix()** function from the R package *caret*.^40^ For each pair of algorithms, the analysis was repeated twice, such that each variable of the pair served once as the reference and once as the predictor. The following measures were used to evaluate pairwise concordance between different algorithms for probable PDD: 1) Cohen’s *κ* with its 95% confidence interval (CI) computed via the **cohen.kappa()** function from the R package *psych*;^41^ 2) Accuracy (i.e., the proportion of correct predictions, both true positives and true negatives, among the total number of cases) with its 95% CI; 3) Sensitivity/Recall (i.e., the proportion of true positives); and 4) Specificity (i.e., the proportion of true negatives).

To assess whether the observed accuracy exceeded what would be expected from trivial classification, the No Information Rate (NIR) was calculated for each pair of algorithms. NIR is the accuracy that could be obtained by always predicting the majority class, and in our case, it is equivalent to the complement of the PDD rate estimate according to the reference algorithm. The accuracy of prediction was compared to the NIR via a one-sided Exact Binomial Test as implemented by the **binom.test()** R stats function. Reference/predictor pairs associated with p < .05 were considered to show significantly better accuracy than NIR. In other words, for reference/predictor pairs associated with p < .05, we conclude that knowing the probable PDD status according to the predictor algorithm helps to estimate the probable PDD status according to the reference algorithm and the two algorithms thus show substantial concordance.

#### Prediction of level II criteria

The third study objective was addressed in two stages. First, a descriptive analysis examined cases where Level II diagnostic algorithms served as the reference and Level I algorithms as predictors, allowing direct comparison of screening-based and comprehensive diagnostic classifications.

Next, to identify a parsimonious screening approximation of PDD classification at Level II, we applied projection-predictive variable selection^42,43^ as implemented in the R package *projpred*.^44^ This approach first fits a comprehensive Bayesian reference model containing all candidate predictors and then searches for smaller submodels whose predictive performance approaches the reference model. In this way, predictor subsets are selected based on their ability to preserve predictive performance rather than on individual parameters’ significance within the reference model, thus substantially reducing the risk of overfitting.

A Bayesian logistic regression predicting Level II PDD status by all available screening variables served as a reference model (The candidate predictors thus included MMSE Sevens, MMSE Pentagons, MMSE Three words, Verbal Fluency S, Clock Drawing (as scored by7), MoCA Sevens, MoCA Cube, MoCA Five words, Verbal Fluency K, MoCA Clock drawing, MoCA Animal naming and MoCA Abstraction). To mitigate potential risks associated with multicollinearity, the regression parameters were assigned regularize horseshoe priors^45^ as implemented in the **horseshoe()** function from the R package *brms*.^46^

Projection predictive variable selection was then used to derive a model with a smaller predictor set. Predictive performance was measured by the expected log pointwise predictive density (ELPD) estimated via cross-validation. The smallest submodel achieving predictive performance comparable to the reference model was selected as the final screening model. Details of the model specification, selection procedure, and performance criteria are provided in the Appendix.

Following variable selection, the optimal probability threshold for predicting Level II PDD classification was determined using the modified, prevalence-adjusted Youden index proposed by^47^ as implemented in the R package *pROC*.^48^ The prevalence adjustment parameter was varied between 0.1 and 0.5 to reflect a plausible population PDD prevalence.

#### Missing data

Missing data were handled by complete case analysis with respect to PDD classification. In other words, only patients for whom all diagnostic algorithms could be applied were included in analyses.

#### Software

Data wrangling and visualizations were done in the *tidyverse* package^49^ and tables were formatted in the *gt* package.^50^ All analyses were conducted within the R (version 4.5.3) software environment for statistical computing.^51^ The software code supporting this article is available at https://github.com/josefmana/demcrit.git.

## Results

### Sample description

A total of 203 patients were considered for the study, out of which thirteen patients were excluded due to missing neuropsychological data, resulting in a final sample of 190 patients. Demographical, clinical and cognitive characteristics of the sample are summarized in Table 2. The sample contained 36 (18.9%) patients with tremor-dominant, 151 (79.5%) patients with akinetic-rigid and 3 (1.6%) patients with axial type of PD. Descriptive statistics for neuropsychiatric symptoms indicated within average levels of depressive and anxiety symptoms in our cohort, with the average BDI-II of 10.71 (SD = 6.97), average STAI X1 of 38.98 (SD = 9.11), and average STAI X2 of 40.23 (SD = 7.80). However, according to the psychiatric assessment, none of the patients with probable PDD was suffering from the major depressive disorder, delirium or other neuropsychiatric abnormalities that would exclude the diagnosis.

**Table 2.** Demographical, clinical and cognitive characteristics of the sample.

	N	Md	Min-max	M	SD
Demographics					
Sex (Males)	122 (64%)	-	-	-	-
Age (in Years)	190	60	34-73	58.82	8.30
Education (in Years)	190	13	8-24	13.81	3.09
Clinical					
Type of PD (Tremor-dominant, Akinetic-rigid, Axial)	36/151/3	-	-	-	-
Hoehn Yahr stage (0-5)	2/7/64/33/14/2	-	-	-	-
PD duration (in Years)	188	10	1-25	10.68	4.19
L-DOPA (in Miligrams)	133	1602	0-4138	1691.80	679.67
UPDRS III off state (Range 0-132)	159	36	10-81	37.33	12.61
UPDRS III on state (Range 0-132)	160	14	1-45	15.79	8.17
MMSE					
Total score (Range 0-30)	190	27	15-30	26.65	2.26
Sevens (Range 0-5)*^1^*	1/2/8/19/32/128	-	-	-	-
VF S (Number of Words per Minute)*^1^*	190	15	1-34	15.04	5.93
Clock Drawing (Range 0-2)	25/86/79	-	-	-	-
Pentagons (Range 0-1)	174 (92%)	-	-	-	-
Three words (Range 0-3)	5/14/52/119	-	-	-	-
MoCA					
Total score (Range 0-30)	190	24	9-30	23.97	3.54
sMoCA total score (Range 0-16)	190	11	1-16	11.21	2.79
Sevens (Range 0-3)	1/2/28/159	-	-	-	-
VF K (Number of Words per Minute)	190	16	0-29	15.54	5.32
Clock drawing (Range 0-3)	23/79/88	-	-	-	-
Cube drawing (Range 0-1)	155 (82%)	-	-	-	-
Five words (Range 0-5)	67/18/27/34/19/25	-	-	-	-
Animal naming (Range 0-3)	10/180	-	-	-	-
Abstraction (Range 0-2)	7/66/117	-	-	-	-
Affect					
BDI-II (Range 0-63)	190	10	0-34	10.71	6.97
STAI X1 (Range 0-80)	179	38	20-72	38.98	9.11
STAI X2 (Range 0-80)	177	40	22-63	40.23	7.80
IADL					
FAQ (Range 0-30)	190	2	0-25	4.05	4.93
FAQ 9 (Range 0-3)	134/45/10/1	-	-	-	-
Screening					
DRS-II (Range0-144)	188	139	115-144	138.07	5.26
NART (Range 0-50)	188	23	1-48	23.38	12.49
Attention and Working Memory					
TMT-A (Z-score)	190	0.03	-13.37-1.41	-0.23	1.31
WAIS DSB (Z-score)	190	-0.14	-2.37-3.23	-0.09	1.09
Executive Function					
CF-A (Z-score)	188	-0.01	-3.47-2.57	-0.07	1.17
PST-C (Z-score)	190	0.17	-6.78-2.05	-0.02	1.00
Language					
WAIS Similarities (Z-score)	190	0.43	-4.87-3.19	0.16	1.82
BNT-60 (with Deficit)	25 (13%)	-	-	-	-
Memory					
RAVLT-DR (Z-score)	190	-0.43	-2.67-2.12	-0.37	0.92
BVMTR-DR (Z-score)	165	3.12	-3.43-9.27	2.99	2.75
WMS-III Family Pictures (Z-score)	23	-0.29	-2.44-0.80	-0.42	0.85
Visuospatial Function					
JoL (Z-score)	190	-0.66	-5.17-1.76	-0.81	1.41
CLOX-I (Z-score)	188	-0.99	-9.26-1.07	-1.45	1.68

*^1^* Not contained within the MMSE but were used by Dubois et al.^7^ in their MMSE-based Level I algorithm for probable PDD.

Note. UPDRS III off state: Unified Parkinson’s Disease Rating Scale Part III, Off medication, UPDRS III on state: Unified Parkinson’s Disease Rating Scale Part III, On medication, BDI-II: Beck Depression Inventory, STAI X1: State Trait-Anxiety Inventory (STAI), Part 1 (state anxiety), STAI X2: STAI, Part 2 (trait anxiety), FAQ: Functional Assessment Questionnaire, FAQ 9: Functional Assessment Questionnaire, Item 9, DRS-II: Dementia Rating Scale Second Edition, NART: National Adult Reading Test, TMT-A: Trail Making Test, Part A, WAIS DSB: Wechsler Adult Intelligence Scale Digit Span, Backwards, CF-A: Categorical Verbal Fluency, Animals, PST-C: Prague Stroop Test, Colours, WAIS Similarities: Wechsler Adult Intelligence Scale, Similarities, BNT-60: Boston Naming Test, RAVLT-DR: Rey Auditory Verbal Learning Test, Delayed Recall, BVMTR-DR: Brief Visuospatial Memory Test, Delayed Recall, WMS-III Family Pictures: Wechsler Memory Scale Family Pictures, JoL: Boston Judgement of Line Orientation, CLOX-I: Clock Drawing Test, all percentages were calculated from the whole sample.

### Dementia rate estimates

Algorithm-wise rate of PDD estimates is presented in Table A3. On average, the estimated PDD rate was 6.21% (SD = 3.43, Md = 4.21, range 2.11-16.84). Notably, the estimates were substantially lower when FAQ item 9 was used as a criterion of IADL deficit (M = 3.31% SD = 0.51, Md = 3.16, range 2.11-4.21) compared to using the total FAQ score criterion (M = 9.10% SD = 2.53, Md = 9.21, range 3.68-16.84) as demonstrated in Figure 1 (see also Figure A4 for per-algorithm distribution of PDD rate estimates). Neither age, sex nor their interaction (*p*s ≥ .101) reliably predicted probable PDD classification across algorithms (see Figure A5 and Figure A6).

### Concordance between algorithms

**Figure 2.**
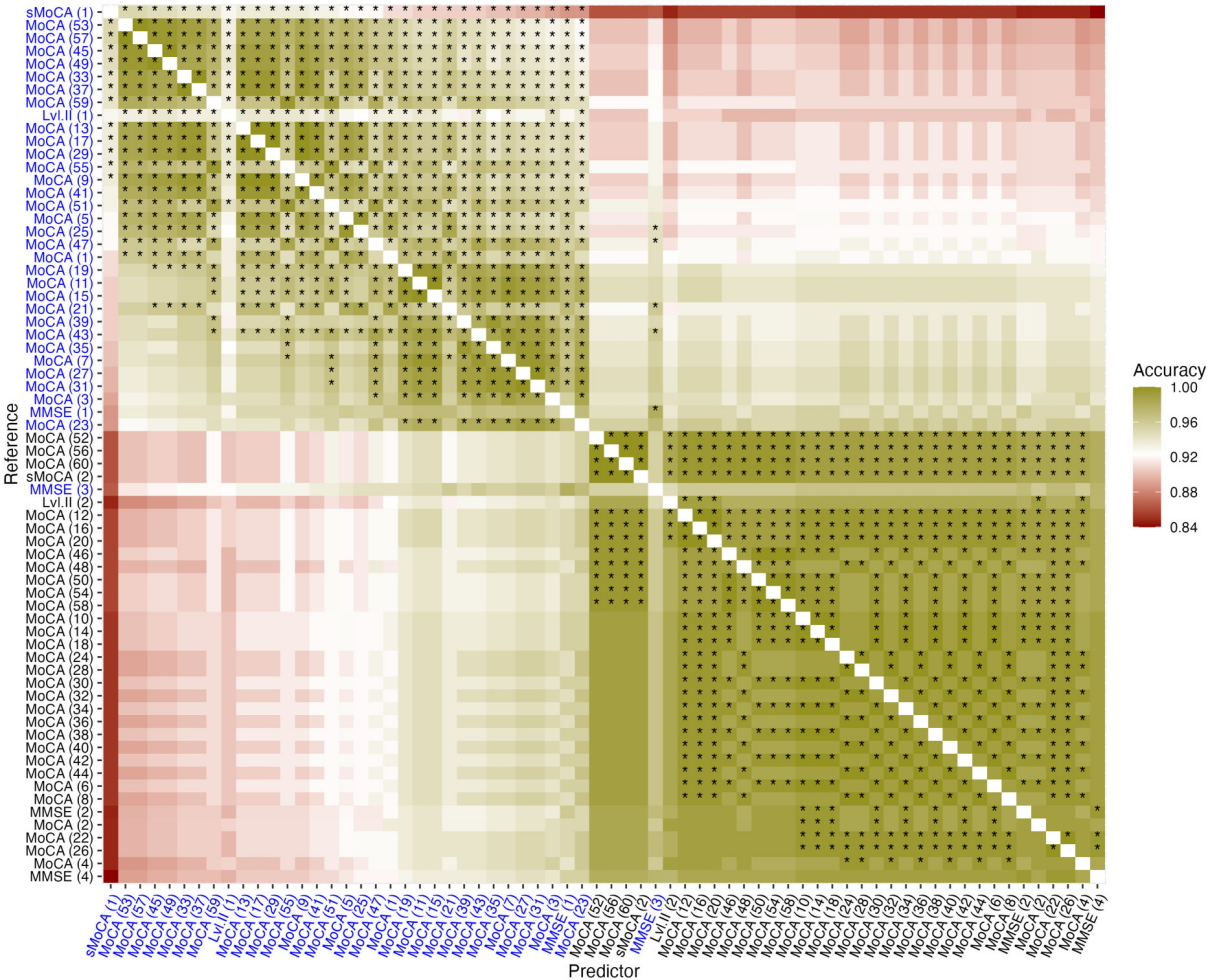
Prediction accuracy matrix. Note. The matrix depicts classification accuracy of algorithms for PDD depicted on x-axis in predicting outcomes based on algorithms on the y-axis. Algorithms printed in blue defined IADL deficit by FAQ total score, algorithms printed in black defined IADL deficit by FAQ item 9 response. Cases with asterisk indicate predictive accuracy statistically significantly higher than the No Information Rate.

Results of the analyses of prediction Accuracy, Cohen’s *κ*, Sensitivity and Specificity are presented in Figure 2, Figure A7, Figure A8 and Figure A9 respectively (Due to the large number of entries (4624 rows x 21 columns representing pairwise comparisons and metrics of interest respectively), the table with numerical results is not presented here or in the Appendix. Instead, we share the table share as data in the accompanying R package available at https://github.com/josefmana/demcrit.git. To obtain the table in format not dependent on R, follow the tutorial at https://josefmana.github.io/demcrit/articles/concordance.html). Generally, algorithms that employed the same operationalization of IADL deficit showed substantial pairwise concordance, however, algorithms that operationalized IADL deficit differently did not. Whereas among algorithms with identical IADL deficit operationalization, the agreement judged by Cohen’s *κ* was moderately high (operationalization by FAQ total score: *κ* = 0.75, SD = 0.14; operationalization by FAQ item 9: *κ* = 0.86, SD = 0.09), among algorithms that differ in IADL deficit operationalization but are otherwise identical it was low: *κ* = 0.43, SD = 0.08.

### Prediction of level II criteria

#### Descriptive analysis

For easier interpretability of our results, we next examined cases where Level II algorithms served as a reference and Level I algorithms as a predictor. Table A4 shows five Level I algorithms with the highest and five with the lowest accuracy in predicting Level II classification of probable PDD. Across IADL deficit definitions, the top five Level I algorithms were all MoCA-based and most of them defined Executive Function deficit by Clock drawing rather than the Verbal fluency test, and Language deficit by Animal naming rather than Abstraction. On the other hand, the MMSE-based criteria performed worse in predicting their respective Level II classification and ranked near the bottom.

However, suppose the predictors are sorted by their balanced accuracy (i.e., average of sensitivity and specificity) instead of raw accuracy. In that case, the results are similar, with the exception that for the prediction of Level II with total FAQ score algorithm for probable PDD, the highest balanced accuracy was achieved by the sMoCA algorithm with sensitivity 0.95 and specificity 0.92 (see Table A5).

#### Exploratory variable selection

Comparisons of submodels’ predictive performance compared to the reference model are shown in Figure A2. When FAQ item 9 was used to define IADL deficit, the intercept-only submodel achieved predictive performance comparable to the reference model. Given the very low PDD rate estimate of this algorithm (3.68%), no further analysis was conducted for this operationalization.

When the FAQ total score was used to define IADL deficit, the projection predictive procedure identified two submodels that matched the predictive performance of the reference model: one containing two predictors and another containing six predictors. Inspection of the ELPD trajectories indicated a clear improvement in predictive performance when the second predictor was added, followed by a decrease in performance for models containing three to five predictors (see Figure A2 A–B). Moreover, because the two-predictor model already achieved good predictive performance and provided substantially greater parsimony, it was selected as the final screening model. The selected model included MoCA Five words and MMSE Sevens as indices of probable PDD (see Figure A3 for ranking of candidate predictors’ importance).

The observed PDD rate according to the Level II algorithm was 10.00%. Screening model optimal decision thresholds according to the prevalence-adjusted Youden criterion are presented in Table 3. These thresholds were clustered according to assumed population prevalence into two groups. For assumed prevalence between 10% and 40%, the optimal threshold corresponded to a predicted PDD probability of 21.5%, yielding a decision rule characterized by high specificity and low sensitivity. Under an assumed prevalence of 50%, the optimal threshold decreased to 6.6%, yielding a decision rule characterized by high sensitivity and low specificity.

**Table 3.** Preliminary clinical scoring rules for approximating Level II probable PDD classification from the selected screening model.

Prevalence	Threshold	Specificity	Sensitivity	Accuracy	Clinical Scoring Rule
0.1-0.4	0.215	0.98	0.32	0.91	MoCA Five words + 1.23 × MMSE Sevens ≤ 3.07
0.5	0.066	0.41	1.00	0.47	MoCA Five words + 1.23 × MMSE Sevens ≤ 8.41

Note. Prevalence: assumed/expected prevalence of Parkinson's disease dementia (PDD); Threshold: optimal threshold of the linear prediction of the screening model on probability scale. Specificity, Sensitivity and Accuracy refer to model performance in predicting PDD according to Level II criteria in current sample. Optimal decision threshold was found using the prevalence-adjusted Youden criterion.

**Figure 3.**
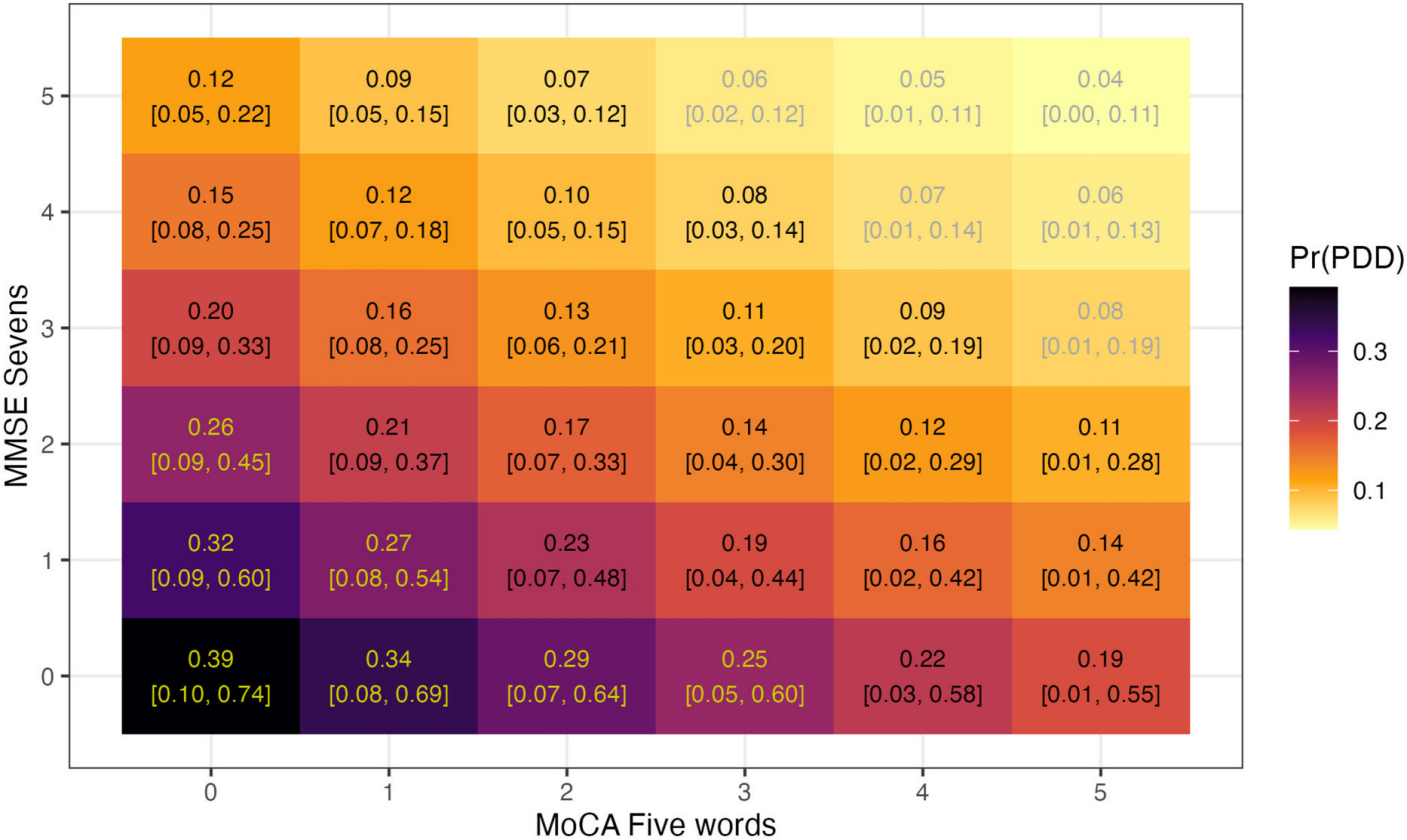
Posterior prediction and decision thresholds for probable PDD based on the selected screening model. Note. The figure shows a heatmap indicating probability of PDD based on posterior prediction of the selected screening model given each possible combination of its predictors. Values in parenthesis indicate 95% equal-tailed posterior probability intervals. Values in yellow indicate performance combinations suggesting probable PDD according to the optimal decision threshold under the assumption of 10-40% prevalence. Values in black indicate performance combinations suggesting probable PDD according to the optimal decision threshold under the assumption of 50% prevalence.

Table 3 translates these model prediction probability thresholds into practical preliminary screening rules expressed as weighted sums of MoCA Five Words and MMSE Sevens raw scores. Moreover, Figure 3 presents posterior predictions of the screening model for all observable combinations of its predictor values together with the corresponding decision thresholds. As evident from the figure, predictive uncertainty increases as the predicted probability of PDD rises.

## Discussion

This study systematically investigated the application of multiple Level I diagnostic algorithms for probable PDD and evaluated their predictive validity with respect to Level II classification. Our results show variability in PDD rate estimates, strongly influenced by the choice of cognitive screening instrument (MMSE, MoCA and sMoCA) and the operationalization of functional impairment. The divergence observed across algorithms demonstrates the sensitivity of diagnostic outcomes to seemingly negligible methodological choices. Predictive validity analyses further indicated that MoCA-based algorithms outperform MMSE-based algorithms and yielded a preliminary two-item screening heuristic that may assist early identification of probable PDD during DBS candidacy evaluation.

### Variability in PDD rate estimates

Our results showed a wide range in estimated PDD rate across algorithms, ranging from 2.11% to 16.84%. Estimates reached lower rates when using solely FAQ item 9 (as an approximation of the pill questionnaire suggested by Dubois et al.^7^) in comparison with the full FAQ scale. This discrepancy highlights the diagnostic importance of how IADLs are assessed.

Our overall PDD rates were consistently lower than previous studies regarding dementia among PD patients, demonstrating wide variability based on various criteria used. For instance, a retrospective study reported a PDD rate of 19.7%,^6^ while other clinical investigations found even higher rates, reaching up to 30%.^52^ A recent meta-analysis synthesizing global data placed the expected PDD rate in PD at 26.30%.^3^ Compared to these estimates, our study reports generally lower PDD rates, likely reflecting differences in diagnostic criteria, methodology and sample characteristics. Specifically, our sample was younger compared to other PD cohorts and age was repeatedly shown to be a strong predictor of PDD across studies.^3,6^

Interestingly, we did not observe any reliable age-related differences in PDD rate within our cohort. This lack of age-dependency may, however, also stem from the relatively younger age of our cohort, because previous reports indicate that the association between age and PDD is not linear but increases with age and may not reach substantial values before older age. In both Rana et al.^6^ and Oh et al.,^5^ nine out of ten patients with dementia were 70 years of age or older. In our sample, only 4.2% participants were in this age range. Consequently, studies with older cohorts are probably necessary to detect a robust association between age and the risk of probable PDD.

### Concordance between diagnostic algorithms

Pairwise comparisons of diagnostic algorithms showed that agreement was notably stronger among those using the same IADL operationalization compared to those using different IADL definitions. Moreover, the agreement was slightly higher between algorithms that defined IADL deficit by FAQ item 9 compared to algorithms that defined it using the full FAQ scale. One possible explanation of this difference follows from the observation that algorithms using the full-scale definition yielded higher PDD rate estimates. Because there was a higher probability of being diagnosed with IADL deficit based on the full FAQ scale, there was also a bigger room for disagreement in the cognitive impairment status when different indexes were used (e.g. by defining executive deficit via Clock Drawing vs. Verbal Fluency).

Overall, when the same IADL definitions were used across algorithms, we observed concordance levels varying from moderate (using FAQ total score) to strong (using FAQ item 9), consistent with inter-rater reliability analysis.^53^ Contrarily, the concordance between algorithms using different IADL deficit definitions was equivalent to minimal agreement. This demonstrates that even slight methodological differences can yield divergent diagnostic outcomes. Such findings are critical for clinicians relying on Level I criteria for eligibility decisions, as the choice of algorithm could lead to contradictory classifications of PDD status.

### Predictive validity comparison with level II criteria

Using Level II diagnosis as the gold standard, MoCA-based Level I algorithms, particularly those using Clock Drawing to assess executive function, demonstrated the highest predictive accuracy. This supports recent proposals to modernize PDD diagnostic frameworks,^9^ favoring MoCA-derived components and more ADL-specific, PD-tailored functional assessment tools. In contrast, MMSE-based algorithms consistently underperformed, suggesting limited sensitivity in capturing cognitive deficits typical in PDD.

Furthermore, in the algorithm using sMoCA, the raw accuracy was moderate, however, the balanced accuracy (i.e. combined sensitivity and specificity) was high. Consequently, sMoCA appears particularly suitable for approximating Level II PDD diagnosis in populations that differ in PDD prevalence from our cohort, since balanced accuracy, unlike raw accuracy, is independent of prevalence in the sample. Moreover, because the sMoCA algorithm demonstrated higher sensitivity while maintaining comparable specificity (see Table A5), it may be especially valuable in contexts where false negatives carry substantial clinical cost. In such cases, a neuropsychologist might use sMoCA as an initial screening tool and proceed to a full Level II assessment only for patients who meet criteria for probable PDD in this preliminary stage.

A further opportunity to simplify screening was explored through a variable selection analysis. When functional impairment was defined using the FAQ total score, a weighted combination of the MoCA Five Words and MMSE Sevens items predicted Level II PDD classification with accuracy comparable to a model including all screening indexes. Because the projection-predictive framework is designed to identify minimal predictive subsets while controlling for overfitting,^42,43^ these findings suggest that a highly parsimonious screening heuristic may be feasible in DBS candidate cohorts. The resulting decision thresholds and posterior predictions are presented in Table 3 and Figure 3.

The variable selection analysis also highlights a practical trade-off between sensitivity and specificity that depends on the assumed prevalence of PDD in the screened population. In practice, clinicians using brief screening tools may need to prioritize either minimising false negatives or limiting unnecessary follow-up assessments. While adding additional screening indexes could improve classification performance, this would increase the complexity and time demands of the screening procedure. Our results suggest that items such as MMSE Pentagons, MoCA Animal Naming, or MoCA Abstraction may represent promising candidates for expanding such parsimonious screening models in DBS candidate cohorts (see Figure A3).

The two items selected for our screening model, MoCA Five Words and MMSE Sevens, may reflect the importance of a fronto-striatal executive deficit in PDD classification within our cohort. Although MoCA Five Words is typically interpreted as a memory measure, it is likely to place greater executive demand than its equivalent in MMSE, the three-words task. Indeed, normative data suggest that MoCA Five Words is the most difficult item of MoCA in Czech neurologically healthy individuals.^54^ Because this item difficulty may partly reflect the longer retention interval rather than executive demands alone, future studies should examine whether the predictive performance of these items persists when administered outside the full MoCA context.

This pattern is consistent with longitudinal cohort studies evidence suggesting that patients considered for DBS represent a subset of the PD population with a distinct cognitive phenotype.^55,56^ Specifically, findings of gradual post-surgical cognitive decline predicted by pre-surgical executive deficits indicate that DBS candidates may be preferentially drawn from a fronto-striatal phenotype characterized by slowly progressing executive dysfunction, rather than from a posterior phenotype marked by visuospatial impairment.^57^ Importantly, patients with the posterior phenotype may be at greater risk of developing PDD within as little as five years after disease onset.^58,59^

### Constraints on generality

This study’s generalizability is limited by the homogeneity of the patient cohort, which does not reflect the diversity of cognitive profiles seen in broader PD populations. Specifically, the younger age of the sample, and a possible underrepresentation of high-risk phenotypes for PDD constrain the generality of the presented findings.

As noted above, the younger age of our sample may partly explain the lower rate of PDD observed compared to previous studies. As discussed in the *Theoretical and Empirical Estimands* section, neither estimates of PDD rate nor predictive performance of demographic variables therefore should be generalized beyond PD patients who are DBS candidates. The extent to which our findings on the concordance between diagnostic algorithms apply to the broader PD population remains to be determined in future studies using different types of cohorts, such as de novo patients or community-based samples.

In our study, visuospatial function was assessed uniformly across all algorithms within a given screening measure (MoCA cube or MMSE pentagons). By contrast, we compared two operationalizations of executive dysfunction, the clock drawing test and verbal fluency. The clock drawing test showed stronger predictive value for level II diagnosis than verbal fluency, suggesting that even within our cohort, patients who developed PDD may exhibit features of the posterior phenotype.

However, the use of a DBS cohort also offered several methodological advantages. All patients underwent standardized and comprehensive neuropsychological testing, resulting in a well-characterized dataset that enabled a systematic evaluation of multiple diagnostic algorithms. Moreover, because dementia is a common exclusion criterion for DBS treatment,^60^ examining the diagnostic accuracy of algorithms for PDD in a pre-DBS cohort is informative in its own right. Moreover, as discussed in the *Theoretical and Empirical Estimands* section, discrepancies in PDD rate estimates arising from alternative definitions of IADL impairment have implications for the construct validity of the diagnostic criteria themselves. If algorithms intended to operationalize the same construct yield divergent classifications when applied to the same patients, this suggests that the construct is not consistently captured by its operational definitions. Such discrepancies, therefore, inform the validity of PDD diagnostic frameworks beyond the specific sample studied.

### Limitations and future directions

Due to the retrospective nature of the study, some patients lacked one or more key measures required for the diagnosis of probable PDD by certain algorithms. Missing data were handled using the complete case method. Although this approach reduces statistical power, it ensures that comparisons between diagnostic algorithms are performed on identical patient subsets, thereby minimizing bias due to sample heterogeneity across pairwise analyses.

An additional limitation concerns the use of the FAQ questionnaire for IADL assessment. The FAQ is a subjective or informant-reliant measure and thus susceptible to bias. Moreover, its content can vary across sociocultural contexts, which limits its cross-cultural transferability. For example, activities such as financial management, cooking or driving, are not universally practiced across societies. Consequently, both the FAQ scores and diagnostic thresholds used to indicate IADL impairment may not be directly transferable between cultural settings.^61^ These factors may influence both the sensitivity and ecological validity of the functional criteria used.^38,62^

Furthermore, IADL measures may correlate with neuropsychological results of specific cognitive domains, particularly attention/processing speed, and executive function.^63,64^ If such correlations were due to shared error variance, they could bias concordance indices measured in this study. To examine this possibility, we conducted a post-hoc simulation experiment, available at https://josefmana.github.io/demcrit/articles/correlation.html. The results indicated that correlations between IADL and neuropsychological measures may either increase or decrease accuracy and balanced accuracy, while consistently inflating Cohen’s *κ* estimates. However, given the correlations observed in our dataset, the effect size was small and unlikely to alter the conclusions of our study.

To address concerns about measuring IADL outlined above, future research should consider using PD-specific questionnaires or more objective tools. Promising options include the Penn Parkinson’s Daily Activities Questionnaire-15,^65^ questionnaire adaptations including items regarding gadget use and digital literacy^66^ or performance-based assessments.^67^ Our findings underscore the importance of IADL measurement for the PDD diagnosis. Therefore, we recommend exploring more reliable tools with high ecological validity.

Another limitation concerns the specific subpopulation included in this study, i.e. patients undergoing evaluation for DBS. As discussed above, this group differs from the broader PD population in several important aspects, including age distribution and cognitive phenotype. Consequently, neither the estimated PDD rates nor the heuristic screening model derived in this study should be generalized to the broader PD population without further validation. Furthermore, the sample included a number of patients at relatively early stages of the disease, characterized by milder clinical severity, shorter disease duration, and lower dopaminergic medication dose. At the same time, the evidence suggests that DBS may also be beneficial in earlier phases of PD,^68^ and the clinical characteristics of our cohort are broadly consistent with those reported in other DBS samples in studies of cognition in PD.^69^ These considerations suggest that, although the findings are not directly generalizable to the broader PD population, they may be relevant to the subpopulation of PD patients considered for DBS.

Additionally, given the retrospective nature of our study, some important sample characteristics, such as the rate of motor fluctuations, dyskinesia or wearing-off impact, were not available. Larger, more heterogeneous cohorts with systematic documentation of these clinical variables would be required to enable approaches such as post-stratification^70^ to overcome this limitation.

Finally, whereas our study systematically investigated how varying definitions of global deficit, impaired cognition and IADL deficit affect probable PDD classification, it did not explore associations of PDD diagnosis with its neuropsychiatric (e.g. anxiety profile) and biomarker correlates. Instead, we only ensured the absence of acute or severe psychiatric symptoms that would preclude a diagnosis of probable PDD, as verified through assessment by a trained neuropsychiatrist. However, both the current diagnostic criteria for PDD^7^ and recent proposals for their revision^9^ emphasize the use of standardized psychometric instruments for assessing neuropsychiatric symptoms. Incorporating such measures could enhance both the efficiency and transparency of the diagnostic process. Future research should therefore investigate how integrating structured neuropsychiatric assessments and biomarker data may refine PDD diagnostic accuracy and improve clinical utility.

## Conclusions

In sum, our study demonstrates that probable PDD classification based on Level I criteria varies substantially depending on how impaired cognition and functional decline are operationalized. In particular, the choice of IADL impairment definition and cognitive screening tool strongly influences diagnostic outcomes. Conservative criteria, such as reliance on pill questionnaire (i.e. FAQ item 9 equivalence), may fail to detect functional decline and thus under-identify true cases of PDD. Importantly, concordance across algorithms rises significantly, reaching moderate to high values, when the same definition of IADL is used (either FAQ total or FAQ item 9). The findings support the call for a change of the current diagnostic criteria,^9^ favoring the use of MoCA-based components and comprehensive IADL assessments.

Within DBS candidate cohorts, Level II classification may be approximated using a highly parsimonious screening combination of MoCA Five Words and MMSE Sevens, although this approach necessarily involves a trade-off between sensitivity and specificity. Future studies should replicate these findings in larger and more diverse PD cohorts and further examine how alternative operationalizations of other diagnostic components, including neuropsychiatric symptoms, affect PDD classification and concordance between algorithms. To make this process easier, the code used to generate our results is publicly available and easily applicable to similarly structured data.

## Supplemental Material

sj-docx-1-pkn-10.1177_1877718X261450361 - Supplemental material for Evaluation of diagnostic concordance between algorithms for Parkinson’s disease dementia Supplemental material, sj-docx-1-pkn-10.1177_1877718X261450361 for Evaluation of diagnostic concordance between algorithms for Parkinson’s disease dementia by Martina Mana, Josef Mana, Petra Stofanikova, Tereza Uhrova, Robert Jech and Ondrej Bezdicek in Journal of Parkinson's Disease
