# Supplementary material for: Evaluation of diagnostic concordance between algorithms for Parkinson’s disease dementia
Source: J Parkinsons Dis. 2026 Jun 11:1877718X261450361. Online ahead of print. doi: 10.1177/1877718X261450361 (PMC13435195; doi:10.1177/1877718X261450361)
Supplement: Supplementary file 1 [file sj-docx-1-pkn-10.1177_1877718X261450361.docx]

# Appendix

## Derivation of the Algorithms Set

Both the original PDD criteria^7^ and the call for their change^9^ allow for several distinct combinations of items to be used to define cognitive impairment. Consequently, in this study, we derived all algorithms for probable PDD on Level I that are in line with published criteria. This procedure parallels the diagnostic algorithm outlined in Table 2 of^7^. Specifically, in this study, we varied the exact specification of items 3-5 of this table (i.e., the measure of global cognitive impairment, the measure of the impact on IADLs and the measures of impaired cognition).

For each set of criteria (MMSE-based, MoCA-based, sMoCA-based and Level II), we first specified the items and then the thresholds for each item used to define probable PDD. If more than one option was present in either the choice of the item or the choice of the threshold, we created an algorithm for each choice in turn. The final set of algorithms was arrived at by computing the Cartesian product of all possibilities provided by varying items and thresholds. All combinations are presented in Table A1.

For MMSE-based algorithms, the following sets of items served as the basis:

$$Global=\{MMSE<26\}$$

$$Attention=\{Sevens backwards<4\}$$

$$Executive=\{Clock drawing<2,Lexical fluency \left( S \right)<10\}$$

$$Construction=\{Pentagons<1\}$$

$Memory = \{{Three{\text-}words\ recall} < 3\}$

$$IADL=\{FAQ>7,FAQ \left( it.9 \right)>1\}$$

The ensuing Cartesian product $Global\times Attention\times Executive\times Construction\times Memory\times IADL$ results in $1\times1\times2\times1\times1\times2=4$ MMSE-based algorithms for probable PDD.

For MoCA-based algorithms, the following sets of items served as the basis:

$$Global=\{MoCA<26\}$$

$$Attention=\{Sevens backwards<3\}$$

$$Executive=\{Clock drawing<2,Clock drawing<3,Lexical fluency \left( K \right)<11\}$$

$$Construction=\{Cube drawing<1\}$$

$Memory = \{Five{\text-}words\ recall < 1, Five{\text-}words\ recall < 2, Five{\text-}words\ recall < 3, Five{\text-}words\ recall < 4, Five{\text-}words\ recall < 5\}$

$$Language=\{Abstraction<2,Animal naming<3\}$$

$$IADL=\{FAQ>7,FAQ \left( it.9 \right)>1\}$$

Note that the additional language domain adds complexity to establishing a diagnostic algorithm because simply by adding it to the set of items, the number of potential algorithms doubles. Further complexity is added by the fact that there are so far no guidelines for selecting a diagnostic threshold for Clock drawing and Five-words recall tests, both of which differ from their counterparts used by^7^. Finally, although the Sevens backwards item has different thresholds in MoCA-based compared to MMSE-based algorithms, this difference is solely due to a difference in scoring whereby 3 points in MoCA correspond to 4 or 5 points in MMSE. The Seven backwards item threshold for MoCA-based algorithms used in this study is thus equivalent to its MMSE-based counterpart.

Computing the Cartesian product $Global\times Attention\times Executive\times Construction\times Memory\times Language\times IADL$ yields $1\times1\times3\times1\times5\times2\times2=60$ distinct MoCA-based algorithms for probable PDD.

For sMoCA-based algorithms, the following sets of items served as the basis:

$$Global=\{sMoCA<13\}$$

$$IADL=\{FAQ>7,FAQ \left( it.9 \right)>1\}$$

yielding $Global\times IADL$, i.e., $1\times2=2$ distinct sMoCA-based algorithms for probable PDD.

Finally, the Level II algorithms were based on the following sets of items:

$$Attention=\{z\left( \text{TMT-A} \right)<-1.5 \cup z\left( WAIS DSB \right)<-1.5\}$$

$$Executive=\{z\left( CF A \right)<-1.5 \cup z\left( \text{PST-C} \right)<-1.5\}$$

$$Construction=\{z\left( JoL \right)<-1.5 \cup z\left( \text{CLOX-I} \right)<-1.5\}$$

$Memory = \{z(\textit{RAVLT-DR}) < -1.5\ \cup z(\textit{BVMT-R- DR}) < -1.5\ \cup z(WMS\text-III\ Family\ Pictures) < -1.5\}$

$$Language=\{z\left( WAIS Similarities \right)<-1.5 \cup z\left( \text{BNT-60} \right)<-1.5\}$$

$$IADL=\{FAQ>7,FAQ \left( it.9 \right)>1\}$$

where $z\left( \right)$ denotes calculation of age, sex and education adjusted z-score. This yields $1\times1\times1\times1\times1\times2=2$ distinct Level II algorithms for probable PDD in the current study. All but the BNT 60 item were evaluated using regression norms published by^11^. Since the original article used BNT 30 instead of BNT 60, we approximated the deficit in BNT 60 by comparing patients’ raw score to age- and education-specific normative values reported by^21^. Specifically, patients whose BNT 60 score fell below the 5^th^ percentile of their demographic group in Table 6 of^21^ were considered to show signs of impaired performance.

### Operationalization of Impaired Cognition

In the original criteria, item 4 of Level I criteria, i.e., impaired cognition, was defined as follows: *“The proposed diagnostic criteria require a profile of cognitive deficits, typical of those described for PD-D, in two or more of four domains.”*^7^ Consequently, we defined impaired cognition as a deficit in two or more domains of four in MMSE-based criteria and as a deficit in two or more of five domains in MoCA-based criteria. sMoCA-based criteria omitted the “impaired cognition” item altogether because they were intended as a shorter screening alternative to classical Level I assessment. Finally, for the Level II criteria, we employed the standard definition of impaired cognition as the *“[i]mpairment on at least two neuropsychological tests, represented by either two impaired tests in one cognitive domain or one impaired test in two different cognitive domains.”*^10^^, Table 1^

Table A1

Summary of all algorithms for probable PDD used in the study.

|  |  | Impaired cognition | | | | |  |
| --- | --- | --- | --- | --- | --- | --- | --- |
| Algorithm | Global deficit | Attention | Executive function | Construction | Memory | Language | Impact on IADLs |
| Lvl.II (1) | - | TMT A < -1.5 OR WAIS DS < -1.5 | CF A < -1.5 OR PST C < -1.5 | JoLO < -1.5 OR CLOXI < -1.5 | RAVLT-DR < -1.5 OR BVMT-R-DR < -1.5 OR WMS-III Family Pictures < -1.5 | WAIS Similarities < -1.5 OR BNT 60 < -1.5 | FAQ > 7 |
| Lvl.II (2) | - | TMT A < -1.5 OR WAIS DS < -1.5 | CF A < -1.5 OR PST C < -1.5 | JoLO < -1.5 OR CLOXI < -1.5 | RAVLT-DR < -1.5 OR BVMT-R-DR < -1.5 OR WMS-III Family Pictures < -1.5 | WAIS Similarities < -1.5 OR BNT 60 < -1.5 | FAQ 9 > 1 |
| MMSE (1) | Total score < 26 | Sevens < 4 | Clock Drawing < 2 | Pentagons < 1 | Three words < 3 | - | FAQ > 7 |
| MMSE (2) | Total score < 26 | Sevens < 4 | Clock Drawing < 2 | Pentagons < 1 | Three words < 3 | - | FAQ 9 > 1 |
| MMSE (3) | Total score < 26 | Sevens < 4 | VF S < 10 | Pentagons < 1 | Three words < 3 | - | FAQ > 7 |
| MMSE (4) | Total score < 26 | Sevens < 4 | VF S < 10 | Pentagons < 1 | Three words < 3 | - | FAQ 9 > 1 |
| MoCA (1) | Total score < 26 | Sevens < 3 | Clock drawing < 2 | Cube drawing < 1 | Five words < 1 | Abstraction < 2 | FAQ > 7 |
| MoCA (10) | Total score < 26 | Sevens < 3 | Clock drawing < 2 | Cube drawing < 1 | Five words < 3 | Abstraction < 2 | FAQ 9 > 1 |
| MoCA (11) | Total score < 26 | Sevens < 3 | Clock drawing < 2 | Cube drawing < 1 | Five words < 3 | Animal naming < 3 | FAQ > 7 |
| MoCA (12) | Total score < 26 | Sevens < 3 | Clock drawing < 2 | Cube drawing < 1 | Five words < 3 | Animal naming < 3 | FAQ 9 > 1 |
| MoCA (13) | Total score < 26 | Sevens < 3 | Clock drawing < 2 | Cube drawing < 1 | Five words < 4 | Abstraction < 2 | FAQ > 7 |
| MoCA (14) | Total score < 26 | Sevens < 3 | Clock drawing < 2 | Cube drawing < 1 | Five words < 4 | Abstraction < 2 | FAQ 9 > 1 |
| MoCA (15) | Total score < 26 | Sevens < 3 | Clock drawing < 2 | Cube drawing < 1 | Five words < 4 | Animal naming < 3 | FAQ > 7 |
| MoCA (16) | Total score < 26 | Sevens < 3 | Clock drawing < 2 | Cube drawing < 1 | Five words < 4 | Animal naming < 3 | FAQ 9 > 1 |
| MoCA (17) | Total score < 26 | Sevens < 3 | Clock drawing < 2 | Cube drawing < 1 | Five words < 5 | Abstraction < 2 | FAQ > 7 |
| MoCA (18) | Total score < 26 | Sevens < 3 | Clock drawing < 2 | Cube drawing < 1 | Five words < 5 | Abstraction < 2 | FAQ 9 > 1 |
| MoCA (19) | Total score < 26 | Sevens < 3 | Clock drawing < 2 | Cube drawing < 1 | Five words < 5 | Animal naming < 3 | FAQ > 7 |
| MoCA (2) | Total score < 26 | Sevens < 3 | Clock drawing < 2 | Cube drawing < 1 | Five words < 1 | Abstraction < 2 | FAQ 9 > 1 |
| MoCA (20) | Total score < 26 | Sevens < 3 | Clock drawing < 2 | Cube drawing < 1 | Five words < 5 | Animal naming < 3 | FAQ 9 > 1 |
| MoCA (21) | Total score < 26 | Sevens < 3 | VF K < 11 | Cube drawing < 1 | Five words < 1 | Abstraction < 2 | FAQ > 7 |
| MoCA (22) | Total score < 26 | Sevens < 3 | VF K < 11 | Cube drawing < 1 | Five words < 1 | Abstraction < 2 | FAQ 9 > 1 |
| MoCA (23) | Total score < 26 | Sevens < 3 | VF K < 11 | Cube drawing < 1 | Five words < 1 | Animal naming < 3 | FAQ > 7 |
| MoCA (24) | Total score < 26 | Sevens < 3 | VF K < 11 | Cube drawing < 1 | Five words < 1 | Animal naming < 3 | FAQ 9 > 1 |
| MoCA (25) | Total score < 26 | Sevens < 3 | VF K < 11 | Cube drawing < 1 | Five words < 2 | Abstraction < 2 | FAQ > 7 |
| MoCA (26) | Total score < 26 | Sevens < 3 | VF K < 11 | Cube drawing < 1 | Five words < 2 | Abstraction < 2 | FAQ 9 > 1 |
| MoCA (27) | Total score < 26 | Sevens < 3 | VF K < 11 | Cube drawing < 1 | Five words < 2 | Animal naming < 3 | FAQ > 7 |
| MoCA (28) | Total score < 26 | Sevens < 3 | VF K < 11 | Cube drawing < 1 | Five words < 2 | Animal naming < 3 | FAQ 9 > 1 |
| MoCA (29) | Total score < 26 | Sevens < 3 | VF K < 11 | Cube drawing < 1 | Five words < 3 | Abstraction < 2 | FAQ > 7 |
| MoCA (3) | Total score < 26 | Sevens < 3 | Clock drawing < 2 | Cube drawing < 1 | Five words < 1 | Animal naming < 3 | FAQ > 7 |
| MoCA (30) | Total score < 26 | Sevens < 3 | VF K < 11 | Cube drawing < 1 | Five words < 3 | Abstraction < 2 | FAQ 9 > 1 |
| MoCA (31) | Total score < 26 | Sevens < 3 | VF K < 11 | Cube drawing < 1 | Five words < 3 | Animal naming < 3 | FAQ > 7 |
| MoCA (32) | Total score < 26 | Sevens < 3 | VF K < 11 | Cube drawing < 1 | Five words < 3 | Animal naming < 3 | FAQ 9 > 1 |
| MoCA (33) | Total score < 26 | Sevens < 3 | VF K < 11 | Cube drawing < 1 | Five words < 4 | Abstraction < 2 | FAQ > 7 |
| MoCA (34) | Total score < 26 | Sevens < 3 | VF K < 11 | Cube drawing < 1 | Five words < 4 | Abstraction < 2 | FAQ 9 > 1 |
| MoCA (35) | Total score < 26 | Sevens < 3 | VF K < 11 | Cube drawing < 1 | Five words < 4 | Animal naming < 3 | FAQ > 7 |
| MoCA (36) | Total score < 26 | Sevens < 3 | VF K < 11 | Cube drawing < 1 | Five words < 4 | Animal naming < 3 | FAQ 9 > 1 |
| MoCA (37) | Total score < 26 | Sevens < 3 | VF K < 11 | Cube drawing < 1 | Five words < 5 | Abstraction < 2 | FAQ > 7 |
| MoCA (38) | Total score < 26 | Sevens < 3 | VF K < 11 | Cube drawing < 1 | Five words < 5 | Abstraction < 2 | FAQ 9 > 1 |
| MoCA (39) | Total score < 26 | Sevens < 3 | VF K < 11 | Cube drawing < 1 | Five words < 5 | Animal naming < 3 | FAQ > 7 |
| MoCA (4) | Total score < 26 | Sevens < 3 | Clock drawing < 2 | Cube drawing < 1 | Five words < 1 | Animal naming < 3 | FAQ 9 > 1 |
| MoCA (40) | Total score < 26 | Sevens < 3 | VF K < 11 | Cube drawing < 1 | Five words < 5 | Animal naming < 3 | FAQ 9 > 1 |
| MoCA (41) | Total score < 26 | Sevens < 3 | Clock drawing < 3 | Cube drawing < 1 | Five words < 1 | Abstraction < 2 | FAQ > 7 |
| MoCA (42) | Total score < 26 | Sevens < 3 | Clock drawing < 3 | Cube drawing < 1 | Five words < 1 | Abstraction < 2 | FAQ 9 > 1 |
| MoCA (43) | Total score < 26 | Sevens < 3 | Clock drawing < 3 | Cube drawing < 1 | Five words < 1 | Animal naming < 3 | FAQ > 7 |
| MoCA (44) | Total score < 26 | Sevens < 3 | Clock drawing < 3 | Cube drawing < 1 | Five words < 1 | Animal naming < 3 | FAQ 9 > 1 |
| MoCA (45) | Total score < 26 | Sevens < 3 | Clock drawing < 3 | Cube drawing < 1 | Five words < 2 | Abstraction < 2 | FAQ > 7 |
| MoCA (46) | Total score < 26 | Sevens < 3 | Clock drawing < 3 | Cube drawing < 1 | Five words < 2 | Abstraction < 2 | FAQ 9 > 1 |
| MoCA (47) | Total score < 26 | Sevens < 3 | Clock drawing < 3 | Cube drawing < 1 | Five words < 2 | Animal naming < 3 | FAQ > 7 |
| MoCA (48) | Total score < 26 | Sevens < 3 | Clock drawing < 3 | Cube drawing < 1 | Five words < 2 | Animal naming < 3 | FAQ 9 > 1 |
| MoCA (49) | Total score < 26 | Sevens < 3 | Clock drawing < 3 | Cube drawing < 1 | Five words < 3 | Abstraction < 2 | FAQ > 7 |
| MoCA (5) | Total score < 26 | Sevens < 3 | Clock drawing < 2 | Cube drawing < 1 | Five words < 2 | Abstraction < 2 | FAQ > 7 |
| MoCA (50) | Total score < 26 | Sevens < 3 | Clock drawing < 3 | Cube drawing < 1 | Five words < 3 | Abstraction < 2 | FAQ 9 > 1 |
| MoCA (51) | Total score < 26 | Sevens < 3 | Clock drawing < 3 | Cube drawing < 1 | Five words < 3 | Animal naming < 3 | FAQ > 7 |
| MoCA (52) | Total score < 26 | Sevens < 3 | Clock drawing < 3 | Cube drawing < 1 | Five words < 3 | Animal naming < 3 | FAQ 9 > 1 |
| MoCA (53) | Total score < 26 | Sevens < 3 | Clock drawing < 3 | Cube drawing < 1 | Five words < 4 | Abstraction < 2 | FAQ > 7 |
| MoCA (54) | Total score < 26 | Sevens < 3 | Clock drawing < 3 | Cube drawing < 1 | Five words < 4 | Abstraction < 2 | FAQ 9 > 1 |
| MoCA (55) | Total score < 26 | Sevens < 3 | Clock drawing < 3 | Cube drawing < 1 | Five words < 4 | Animal naming < 3 | FAQ > 7 |
| MoCA (56) | Total score < 26 | Sevens < 3 | Clock drawing < 3 | Cube drawing < 1 | Five words < 4 | Animal naming < 3 | FAQ 9 > 1 |
| MoCA (57) | Total score < 26 | Sevens < 3 | Clock drawing < 3 | Cube drawing < 1 | Five words < 5 | Abstraction < 2 | FAQ > 7 |
| MoCA (58) | Total score < 26 | Sevens < 3 | Clock drawing < 3 | Cube drawing < 1 | Five words < 5 | Abstraction < 2 | FAQ 9 > 1 |
| MoCA (59) | Total score < 26 | Sevens < 3 | Clock drawing < 3 | Cube drawing < 1 | Five words < 5 | Animal naming < 3 | FAQ > 7 |
| MoCA (6) | Total score < 26 | Sevens < 3 | Clock drawing < 2 | Cube drawing < 1 | Five words < 2 | Abstraction < 2 | FAQ 9 > 1 |
| MoCA (60) | Total score < 26 | Sevens < 3 | Clock drawing < 3 | Cube drawing < 1 | Five words < 5 | Animal naming < 3 | FAQ 9 > 1 |
| MoCA (7) | Total score < 26 | Sevens < 3 | Clock drawing < 2 | Cube drawing < 1 | Five words < 2 | Animal naming < 3 | FAQ > 7 |
| MoCA (8) | Total score < 26 | Sevens < 3 | Clock drawing < 2 | Cube drawing < 1 | Five words < 2 | Animal naming < 3 | FAQ 9 > 1 |
| MoCA (9) | Total score < 26 | Sevens < 3 | Clock drawing < 2 | Cube drawing < 1 | Five words < 3 | Abstraction < 2 | FAQ > 7 |
| sMoCA (1) | sMoCA total score < 13 | - | - | - | - | - | FAQ > 7 |
| sMoCA (2) | sMoCA total score < 13 | - | - | - | - | - | FAQ 9 > 1 |
| Note. UPDRS III off state: Unified Parkinson’s Disease Rating Scale Part III, Off medication, UPDRS III on state: Unified Parkinson’s Disease Rating Scale Part III, On medication, BDI-II: Beck Depression Inventory, STAI X1: State Trait-Anxiety Inventory (STAI), Part 1 (state anxiety), STAI X2: STAI, Part 2 (trait anxiety), FAQ: Functional Assessment Questionnaire, FAQ 9: Functional Assessment Questionnaire, Item 9, DRS-II: Dementia Rating Scale Second Edition, NART: National Adult Reading Test, TMT-A: Trail Making Test, Part A, WAIS DSB: Wechsler Adult Intelligence Scale Digit Span, Backwards, CF-A: Categorical Verbal Fluency, Animals, PST-C: Prague Stroop Test, Colors, WAIS Similarities: Wechsler Adult Intelligence Scale, Similarities, BNT-60: Boston Naming Test, RAVLT-DR: Rey Auditory Verbal Learning Test, Delayed Recall, BVMT-R-DR: Brief Verbal Memory Test, Delayed Recall, WMS-III Family Pictures: Wechsler Memory Scale Family Pictures, JoL: Boston Judgement of Line Orientation, CLOX-I: Clock Drawing Test | | | | | | | |

## Theoretical and Empirical Estimands Elaborated

In this study, we follow the framework proposed by^39^ for specifying targets of inference (i.e., the estimands) in quantitative sciences to increase transparency and connect statistical evidence to relevant theory. Table A2 contains a verbal description of the components relating to each of our proclaimed research objectives and maps them to the population quantity of interest (the theoretical estimand), data-dependent quantity that could be estimated (the empirical estimand) and quantities that are reported in the study (statistical estimates).

Table A2

Mapping between research objectives and quantities of interest in the current study.

|  | Theoretical Estimand | |  |  |
| --- | --- | --- | --- | --- |
|  | Quantity | Population | Empirical Estimand | Statistical Estimate |
| Research Objective 1 | | | | |
| To estimate the rate of PDD within PD. | The true rate of PDD within the population of patients with PD. | DBS candidates with PD | Proportion of patients classified as having probable PDD according to each of the algorithms under consideration. | Measures of central tendency (mean, median) of the proportion of patients with probable PDD in the sample across algorithms under consideration. |
| To assess the variability of PDD diagnosis depending on the algorithm applied. | The variance of PDD rate estimates is attributable to the diagnostic process itself, i.e., the extent to which different algorithms for probable PDD yield differing PDD rate estimates for the same population. | DBS candidates with PD | Shape of the distribution of probable PDD rate according to each of the algorithms under consideration. | Measures of variability (standard deviation, range) of the proportion of patients with probable PDD in the sample across algorithms under consideration. |
| To evaluate predictive information provided by demographic variables for probable PDD diagnosis. | The expected change in of suffering with PDD given a unit change in age or sex. | DBS candidates with PD | Estimates of expected change in the expected value of the probability of PDD given a change in age or sex for each of the algorithms under consideration. | A set of transformed logistic regression coefficients (Odds Ratios) for age, sex and their statistical interaction for each of the algorithms under consideration. |
| To evaluate the diagnostic concordance between different PDD algorithms within and between PDD criteria. | A set of population-level contingency tables comparing probable PDD classification assigned by each pair of probable PDD algorithms, along with derived metrics such as sensitivity, specificity, and inter-algorithm reliability coefficients. | General population of patients with PD | Matrices generated by pairwise Receiver Operating Characteristic (ROC) curve analyses that evaluate the discriminatory performance of each probable PDD algorithm. | Metrics derived from pairwise ROC analyses - Cohen's Kappa, Sensitivity, Specificity, Accuracy and statistical comparison between Accuracy and No Information Rate (NIR). |
| Research Objective 2 | | | | |
| To identify the algorithms' components that contribute to variability in probable PDD diagnosis within and across criteria. | A set of algorithms' components (such as diagnostic thresholds or test indexes being used to measure a certain cognitive domain) whose variation systematically alters the probability of a probable PDD diagnosis. | General population of patients with PD | Changes in the proportion of probable PDD induced by varying sets of algorithm components. | Visually inspected differences in probable PDD rate distributions conditional on sets of algorithms' components. |
| Research Objective 3 | | | | |
| To evaluate the diagnostic concordance between Level I and Level II PDD algorithms. | A set of Level I probable PDD algorithms, ranked by the accuracy in predicting Level II PDD classification. | DBS candidates with PD | Level I probable PDD algorithms ranking based on their accuracy of predicting PDD classification on Level II in the sample. | Ranking of the available algorithms for probable PDD |
| To identify a minimal set of screening indexes that well approximate Level II PDD classification. | A classification model of Level II PDD classification given a few screening indexes from Level I. | DBS candidates with PD | Logistic regression model with reduced prediction set based on projective prediction on a reference model containing all available candidate predictors. | A set of optimal decision equation based on ROC analysis under different assumed real PDD prevalences. |

The RO1 - to estimate the PDD rate and evaluate the diagnostic variability and concordance across different algorithms of probable PDD - was divided into four distinct research objectives:

- to estimate the rate of PDD within PD (RO1.1),
- to estimated variability of this rate (RO1.2),
- to evaluate predictive value of demographic variables for probable PDD classification (RO1.3) and
- to evaluate concordance between different probable PDD operationalizations and criteria (RO1.4).

Estimates relating to RO1.1**-**1.3 cannot be safely generalized beyond a population of PD patients that are candidates for DBS due to the systematic differences between the DBS candidates pool and the general PD population (such as the lower age or different cognitive phenotypes of DBS candidates compared to the general PD population). On the other hand, the estimate relating to RO1.4 (and to a lesser degree to RO1.2) may not be substantially influenced by the sample at hand as the primary source of their variance is likely to come from variability in measures employed (e.g., MMSE vs MoCA to assess global cognitive performance) rather than variability in patients’ performance. Assuming that there is no substantial Differential Item Functioning for DBS candidates compared to a broader population of patients with PD, the estimates relating to RO1.4 can be cautiously generalized beyond the current sample.

For the RO2, the theoretical estimand is defined as the set of diagnostic components whose variation systematically alters the probability of a probable PDD diagnosis. This aspect of the study is exploratory in nature. Empirically, we assess the contribution of each diagnostic feature by examining how variations in operational definitions (e.g., domain-specific thresholds, criteria for functional impairment) influence the statistical estimates derived for the first objective. This allows us to identify the diagnostic elements most responsible for between-algorithm discrepancies.

Finally, for the RO3, we define two sets of theoretical and statistical estimands. The first, descriptive estimand (RO4.1) is defined as a ranking of Level I probable PDD algorithms according to their accuracy in predicting PDD classification at Level II. The second, exploratory predictive estimand (RO3.2) is defined as a classification model for Level II PDD based on a reduced subset of cognitive screening indexes selected from the full candidate predictor set. The statistical estimate for RO3.2 is defined as a projection predictive classification logistic regression model fitted to our sample, and following the derived quantities:

- posterior predictions of PDD probability evaluated across the full grid of possible predictor values (Figure 3),
- optimal decision thresholds for Level II PDD classification under different assumed prevalence values (Table 3).

Importantly, because cognitive profiles of DBS candidates differ from those of the general PD population^55^^,^^56^ and the dependence of RO3 estimates on our sample, the results cannot be safely generalized to the broader population of people with PD. Instead, the estimates relating to RO3 are relevant to a population of PD DBS candidates and could thus be leveraged in the process of DBS candidacy for initial screening for DBS exclusion criteria.

## Projection Predictive Variable Selection Procedure

### Reference Model Specification

To derive a parsimonious set of screening cognitive variables approximating classification of PDD at Level II, we employed projection predictive variable selection^42^^,^^43^.

The procedure begins with fitting a reference model containing all candidate predictors. The reference model used here was a Bayesian logistic regression:

$$y_{i}\sim Bernoulli\left( p_{i} \right)$$

$$logit\left( p_{i} \right)=\beta_{0}+\sum_{j=1}^{K} \beta_{j}x_{ij}$$

$i$ = 1…$N$, where $N$ is the total number of patients, $K$ is the total number of predictors, $y_{i}$ denotes Level II classification for patient $i$ ($y_{i}=1$ if probable PDD is diagnosed, $y_{i}=0$ otherwise) and $x_{ij}$ represents $j$-th screening variable for patient $i$.

The candidate predictors included the following screening indices:

- MMSE sevens,
- MMSE pentagons drawing,
- MMSE three Words,
- Verbal Fluency (S),
- Clock Drawing (^7^ scoring),
- MoCA sevens,
- MoCA cube drawing,
- MoCA five words,
- Verbal Fluency (K),
- MoCA clock drawing,
- MoCA animal naming,
- MoCA abstraction.

Figure A1 shows bivariate correlations between all candidate predictor variables in the sample. Before entering the model, all candidate predictors were scaled by their in-sample means and standard deviations.

Figure A1

Correlation matrix of the candidate predictor pool.

*Note*. Bivariate Spearman correlation coefficients based on the sample of N = 190 patients is plotted. Indexes are ordered according to the assumed cognitive domain.

### Prior Specification

To mitigate risks associated with multicollinearity and overfitting, all regression coefficients barring the intercept were assigned regularized horseshoe priors^42^. Specifically,

$$\beta_{j}\sim Horseshoe\left( \tau,\lambda_{j} \right)$$

where $\lambda_{j}$ represents item-specific local shrinkage parameter and $\tau$ represents the global shrinkage parameter controlling overall sparsity.

The prior was implemented using the horseshoe() function from the *brms* package^46^ with parameters $df=3$ and $par\_ratio=r$, where $r$ reflects the ratio of expected non-zero parameters to the full predictor set. In the present analysis, $r$ was based on 4 or 5 expected non-zero parameters to reflect the expectation that only a minority of screening variables would meaningfully contribute to prediction. Specifically,^7^ define 4 domains to define cognitive impairment on Level I, while^9^ suggest adding a fifth, language domain.

The intercept parameter was assigned uniform prior as per *brms* defaults.

The reference model included four chains, each running for 1,000 warmup (later discarded) iterations, and 2,500 further iterations of a Hamiltonian Monte Carlo (HMC) algorithm, resulting in 10,000 samples from the posterior distribution. The model were fitted using the HMC sampler in Stan (version 2.32.2)^71^

### Projection Predictive Variable Selection

Following estimation of the reference model, projection predictive variable selection was conducted using the R package *projpred*^44^.

The procedure evaluates a sequence of reduced submodels containing progressively smaller subsets of predictors. For each candidate submodel, the predictive distribution is obtained by projecting the reference model posterior onto the reduced model parameter space, ensuring that the reduced submodel approximates the prediction made by the reference model.

Predictive performance was evaluated using the expected log pointwise predictive density (ELPD) estimated via cross-validation using the cv_varsel() function with 20 clusters and 1,000 draws from the posterior.

Model size was determined using the following heuristic with a combination of visual examination of ELPD difference plots: for selection, the smallest submodels were considered for which the upper bound of the 68.3% normal-approximation interval (+/- 1 SD) of the submodel exceeded the ELPD estimate of the reference model.

Results of ELPD comparisons are presented in Figure A2. When the FAQ item 9 score was used to define IADL impairment, none of the predictors was selected by the heuristic (Figure A2 C). On the other hand, under the FAQ total score definition of IADL impairment, either two or six predictors were selected by the heuristic (Figure A2 A and B). The pattern of results was similar for $r=4$ and $r=5$ and the exact results differed between runs of the modelling pipeline due to randomness in the sampling process.

Given the overall pattern of ELPD differences (showing clear improvement when two predictors are selected with clear deterior of predictive performance when selecting three to five predictors), and greater parsimony of two compared to six predictors, the two-predictor classification model was selected for further analysis. Figure A3 shows the cumulative ranking of the predictors based on the number of cross-validation folds in which each predictor was included to progressively smaller submodels.

Figure A2

Expected log pointwise predictive density (ELPD) comparison.

*Note*. The figure shows comparison of submodels’ to reference model’s ELPDs with their 68.3% (i.e., +/- one standard deviation) normal-approximation intervals for operationalizations of IADL deficit by FAQ total score (A and B) and FAQ item 9 (C). The reference models in A and C were fitted expecting 4 non-zero parameters, reference model in B was fitted expecting 5 non-zero parameters. The two-predictor model depicted in A was retained for further analysis.

Figure A3

Cummulative ranking of predictors from the process of selection of the final screening models.

*Note*. The figure shows in what proportion of cross-validation folds were different candidate predictors (ordinate) included during the projective prediction process given submodel size (abscissa). Predictors are order from the least (bottom) to the most (top) important.

### Decision Threshold Estimation

To derive a practical screening rule, the optimal probability threshold for predicting Level II PDD classification was determined using the modified Youden index proposed by^47^. The modification parameter $r_{p}$ was varied between:

$$r_{p}\in\{0.1,0.2,0.3,0.4,0.5\}$$

to reflect plausible population prevalence values for PDD.

Finally, a screening decision equations for each unique optimal threshold were derived by re-weighting the following inequality:

$$\sum_{j=1}^{M} \beta_{j}x_{ij}\leq\left( threshold-\beta_{0} \right)$$

where $M$ is the number of selected predictors by the smallest $\beta_{j}$, i.e., $min\left( \beta\right)$, resulting into a clinical screening rule equation^[1]^:

$$\sum_{j=1}^{M} \frac{\beta_{j}}{min\left( \beta\right)}x_{ij}\leq\frac{\left( threshold-\beta_{0} \right)}{min\left( \beta\right)}$$

.

## Supplementary Presentation of Results

Figure A4

Summary of the estimates of probable PDD rate.

*Note*. Vertical lines represent estimates arrived at by using sMoCA (dotted) or Level II (dashed) with FAQ item 9 (orange) or FAQ total score (blue) as criteria for probable PDD. The percentages were calculated from all available cases (see Table A3 for numerical summary).

Table A3

Estimates of the rate of probable PDD in the sample.

| Algorithm | N | Rate |
| --- | --- | --- |
| sMoCA (1) | 190 | 32 (16.84%) |
| MoCA (53) | 190 | 23 (12.11%) |
| MoCA (57) | 190 | 23 (12.11%) |
| MoCA (45) | 190 | 22 (11.58%) |
| MoCA (49) | 190 | 22 (11.58%) |
| MoCA (33) | 190 | 21 (11.05%) |
| MoCA (37) | 190 | 21 (11.05%) |
| MoCA (59) | 190 | 21 (11.05%) |
| MoCA (13) | 190 | 20 (10.53%) |
| MoCA (17) | 190 | 20 (10.53%) |
| MoCA (29) | 190 | 20 (10.53%) |
| MoCA (55) | 190 | 20 (10.53%) |
| MoCA (9) | 190 | 20 (10.53%) |
| Lvl.II (1) | 190 | 19 (10.00%) |
| MoCA (41) | 190 | 19 (10.00%) |
| MoCA (51) | 190 | 19 (10.00%) |
| MoCA (5) | 190 | 18 (9.47%) |
| MoCA (25) | 190 | 17 (8.95%) |
| MoCA (47) | 190 | 17 (8.95%) |
| MoCA (1) | 190 | 15 (7.89%) |
| MoCA (19) | 190 | 15 (7.89%) |
| MoCA (39) | 190 | 15 (7.89%) |
| MoCA (11) | 190 | 14 (7.37%) |
| MoCA (15) | 190 | 14 (7.37%) |
| MoCA (21) | 190 | 14 (7.37%) |
| MoCA (35) | 190 | 14 (7.37%) |
| MoCA (43) | 190 | 14 (7.37%) |
| MoCA (27) | 190 | 13 (6.84%) |
| MoCA (31) | 190 | 13 (6.84%) |
| MoCA (7) | 190 | 13 (6.84%) |
| MMSE (1) | 190 | 11 (5.79%) |
| MoCA (23) | 190 | 11 (5.79%) |
| MoCA (3) | 190 | 11 (5.79%) |
| MoCA (52) | 190 | 8 (4.21%) |
| MoCA (56) | 190 | 8 (4.21%) |
| MoCA (60) | 190 | 8 (4.21%) |
| sMoCA (2) | 190 | 8 (4.21%) |
| Lvl.II (2) | 190 | 7 (3.68%) |
| MMSE (3) | 190 | 7 (3.68%) |
| MoCA (12) | 190 | 7 (3.68%) |
| MoCA (16) | 190 | 7 (3.68%) |
| MoCA (20) | 190 | 7 (3.68%) |
| MoCA (46) | 190 | 7 (3.68%) |
| MoCA (48) | 190 | 7 (3.68%) |
| MoCA (50) | 190 | 7 (3.68%) |
| MoCA (54) | 190 | 7 (3.68%) |
| MoCA (58) | 190 | 7 (3.68%) |
| MoCA (10) | 190 | 6 (3.16%) |
| MoCA (14) | 190 | 6 (3.16%) |
| MoCA (18) | 190 | 6 (3.16%) |
| MoCA (24) | 190 | 6 (3.16%) |
| MoCA (28) | 190 | 6 (3.16%) |
| MoCA (30) | 190 | 6 (3.16%) |
| MoCA (32) | 190 | 6 (3.16%) |
| MoCA (34) | 190 | 6 (3.16%) |
| MoCA (36) | 190 | 6 (3.16%) |
| MoCA (38) | 190 | 6 (3.16%) |
| MoCA (40) | 190 | 6 (3.16%) |
| MoCA (42) | 190 | 6 (3.16%) |
| MoCA (44) | 190 | 6 (3.16%) |
| MoCA (6) | 190 | 6 (3.16%) |
| MoCA (8) | 190 | 6 (3.16%) |
| MMSE (2) | 190 | 5 (2.63%) |
| MoCA (2) | 190 | 5 (2.63%) |
| MoCA (22) | 190 | 5 (2.63%) |
| MoCA (26) | 190 | 5 (2.63%) |
| MoCA (4) | 190 | 5 (2.63%) |
| MMSE (4) | 190 | 4 (2.11%) |
| Note. Percentages were calculated from all available cases, the items comprising each listed algorithm can be found in Table A1. | | |

Table A4

Level I algorithms for probable PDD as predictors of Level II classification as the reference.

| Level II (1)*^a^* | | | | | | Level II (2)*^b^* | | | | | |
| --- | --- | --- | --- | --- | --- | --- | --- | --- | --- | --- | --- |
| Predictor | κ | Accuracy | p | Sensitivity | Specificity | Predictor | κ | Accuracy | p | Sensitivity | Specificity |
| Top five | | | | | | | | | | | |
| MoCA (23) | 0.62 | 0.94 | .017 | 0.50 | 0.99 | MoCA (12) | 0.85 | 0.99 | .028 | 0.86 | 0.99 |
| MoCA (3) | 0.62 | 0.94 | .017 | 0.50 | 0.99 | MoCA (16) | 0.85 | 0.99 | .028 | 0.86 | 0.99 |
| MoCA (51) | 0.69 | 0.94 | .017 | 0.70 | 0.97 | MoCA (2) | 0.83 | 0.99 | .028 | 0.71 | 1.00 |
| MoCA (11) | 0.61 | 0.94 | .032 | 0.55 | 0.98 | MoCA (20) | 0.85 | 0.99 | .028 | 0.86 | 0.99 |
| MoCA (13) | 0.67 | 0.94 | .032 | 0.70 | 0.97 | MoCA (4) | 0.83 | 0.99 | .028 | 0.71 | 1.00 |
| Bottom five | | | | | | | | | | | |
| MoCA (5) | 0.59 | 0.93 | .093 | 0.60 | 0.97 | MoCA (50) | 0.70 | 0.98 | .168 | 0.71 | 0.99 |
| sMoCA (1) | 0.69 | 0.93 | .093 | 0.95 | 0.92 | MoCA (54) | 0.70 | 0.98 | .168 | 0.71 | 0.99 |
| MMSE (3) | 0.43 | 0.93 | .136 | 0.32 | 0.99 | MoCA (58) | 0.70 | 0.98 | .168 | 0.71 | 0.99 |
| MoCA (25) | 0.55 | 0.92 | .143 | 0.55 | 0.97 | MMSE (2) | 0.66 | 0.98 | .168 | 0.57 | 0.99 |
| MoCA (39) | 0.53 | 0.92 | .143 | 0.50 | 0.97 | MMSE (4) | 0.53 | 0.97 | .296 | 0.43 | 0.99 |
| *^a^*IADL deficit was defined as FAQ (total score) > 7 | | | | | | | | | | | |
| *^b^*IADL deficit was defined as FAQ (item 9) > 1 | | | | | | | | | | | |
| κ: Cohen's κ; p: p-value associated with a one-sided Exact Binomial Test comparing the Accuracy to the No Information Rate; The table shows five most accurate (Top five) and five least accurate (Bottom five) Level I algorithms for Parkinson's Disease Dementia (PDD) in predicting Level II classificiation of PDD. The algorithms were grouped by their definition of the deficit in Instrumental Activities of Daily Living (IADLs). The items comprising each listed algorithm can be found in Table A1. | | | | | | | | | | | |

Table A5

Level I algorithms for probable PDD as predictors of Level II classification as the reference arranged by their balanced accuracy score.

| Level II (1)*^a^* | | | | | | Level II (2)*^b^* | | | | | |
| --- | --- | --- | --- | --- | --- | --- | --- | --- | --- | --- | --- |
| Predictor | κ | Accuracy | p | Sensitivity | Specificity | Predictor | κ | Accuracy | p | Sensitivity | Specificity |
| Top five | | | | | | | | | | | |
| sMoCA (1) | 0.69 | 0.93 | .093 | 0.95 | 0.92 | MoCA (12) | 0.85 | 0.99 | .028 | 0.86 | 0.99 |
| MoCA (45) | 0.68 | 0.94 | .032 | 0.75 | 0.96 | MoCA (16) | 0.85 | 0.99 | .028 | 0.86 | 0.99 |
| MoCA (49) | 0.68 | 0.94 | .032 | 0.75 | 0.96 | MoCA (20) | 0.85 | 0.99 | .028 | 0.86 | 0.99 |
| MoCA (53) | 0.66 | 0.93 | .056 | 0.75 | 0.95 | MoCA (52) | 0.79 | 0.98 | .078 | 0.86 | 0.99 |
| MoCA (57) | 0.66 | 0.93 | .056 | 0.75 | 0.95 | MoCA (56) | 0.79 | 0.98 | .078 | 0.86 | 0.99 |
| Bottom five | | | | | | | | | | | |
| MoCA (21) | 0.55 | 0.93 | .093 | 0.50 | 0.98 | MoCA (58) | 0.70 | 0.98 | .168 | 0.71 | 0.99 |
| MoCA (35) | 0.55 | 0.93 | .093 | 0.50 | 0.98 | MoCA (22) | 0.66 | 0.98 | .168 | 0.57 | 0.99 |
| MoCA (39) | 0.53 | 0.92 | .143 | 0.50 | 0.97 | MoCA (26) | 0.66 | 0.98 | .168 | 0.57 | 0.99 |
| MMSE (1) | 0.55 | 0.93 | .056 | 0.45 | 0.99 | MMSE (2) | 0.66 | 0.98 | .168 | 0.57 | 0.99 |
| MMSE (3) | 0.43 | 0.93 | .136 | 0.32 | 0.99 | MMSE (4) | 0.53 | 0.97 | .296 | 0.43 | 0.99 |
| *^a^*IADL deficit was defined as FAQ (total score) > 7 | | | | | | | | | | | |
| *^b^*IADL deficit was defined as FAQ (item 9) > 1 | | | | | | | | | | | |
| κ: Cohen's κ; p: p-value associated with a one-sided Exact Binomial Test comparing the Accuracy to the No Information Rate; The table shows five most accurate (Top five) and five least accurate (Bottom five) Level I algorithms for Parkinson's Disease Dementia (PDD) in predicting Level II classificiation of PDD. The algorithms were grouped by their definition of the deficit in Instrumental Activities of Daily Living (IADLs). The items comprising each listed algorithm can be found in Table A1. | | | | | | | | | | | |

Figure A5

Representation of study data.

*Note*. The figure shows whether patients (x-axis) ordered from the youngest (left) to the oldest (right) were classified as probable PDD by each tested algorithm (y-axis) ordered from the one with the lowest (bottom) to the highest (top) PDD rate estimate. Patients printed in red are women, patients printed in blue are men. Red cells indicate probable PDD diagnosis, grey cells indicate non-PDD diagnosis and white cells indicate missing diagnosis.

Figure A6

Summary of logistic regressions parameters prediction probable PDD by age and sex.

*Note*. Histograms represent odd ratio (OR) estimates and p-values associated with age, sex, and their interaction as predictors of each of the 68 probable PDD classification. In the case of parameters for sex, values higher than 20 were omitted for clarity. Vertical lines indicate OR = 1 and p = .05.

Figure A7

Cohen’s κ matrix.

*Note*. The matrix depicts Cohen’s κ measuring agreement between algorithms for PDD. Algorithms printed in red defined IADL deficit by FAQ total score, algorithms printed in black defined IADL deficit by FAQ item 9 response.

Figure A8

Sensitivity matrix.

*Note*. The matrix depicts sensitivity of algorithms for PDD depicted on x-axis in predicting outcomes based on algorithms on the y-axis. Algorithms printed in blue defined IADL deficit by FAQ total score, algorithms printed in black defined IADL deficit by FAQ item 9 response.

Figure A9

Specificity matrix.

*Note*. The matrix depicts specificity of algorithms for PDD depicted on x-axis in predicting outcomes based on algorithms on the y-axis. Algorithms printed in blue defined IADL deficit by FAQ total score, algorithms printed in black defined IADL deficit by FAQ item 9 response.

1. Because the predictor variables were scaled before model computations, implementation of this equation included further step of back-transforming parameters to their respective response scales. This step was omitted from the description for conceptual clarity, but it can be checked on the study’s repository [https:://github.com/josefmana/demcrit.git](NULL)
